# Indoor Bacterial and Fungal Burden in “Moldy” versus “Non-Moldy” Homes: A Case Study Employing Advanced Sequencing Techniques in a US Metropolitan Area

**DOI:** 10.3390/pathogens12081006

**Published:** 2023-08-01

**Authors:** Bhavin V. Chauhan, Daleniece Higgins Jones, Goutam Banerjee, Saumya Agrawal, Irshad M. Sulaiman, Chunrong Jia, Pratik Banerjee

**Affiliations:** 1 Division of Epidemiology, Biostatistics, and Environmental Health, School of Public Health, University of Memphis, Memphis, TN 38152, USA; 2 Department of Public Health, University of Tennessee, Knoxville, TN 37996, USA; 3 Department of Food Science and Human Nutrition, University of Illinois at Urbana-Champaign, Urbana, IL 61820, USA; 4 Southeast Regional Laboratory, U.S. Food and Drug Administration, Atlanta, GA 30309, USA

**Keywords:** NGS, fungal communities, bacterial communities, qRT-PCR, indoor environments

## Abstract

The presence of fungi in the indoor environment is associated with allergies and other respiratory symptoms. The aim of this study was to use sequencing and molecular methods, including next-generation sequencing (NGS) approaches, to explore the bacterial and fungal communities and their abundance in the indoor environment of houses (*n* = 20) with visible “moldy” (HVM) and nonvisible “non-moldy” (HNM) in Memphis, TN, USA. Dust samples were collected from air vents and ground surfaces, and the total DNA was analyzed for bacteria and fungi by amplifying 16S rRNA and ITS genes on the Illumina Miseq. Results indicated that *Leptosphaerulina* was the most abundant fungal genus present in the air vent and ground samples from HNM and HVM. At the same time, the most abundant bacterial genera in the air vent and ground samples were *Propionibacterium* and *Streptococcus*. The fungi community diversity was significantly different in the air vent samples. The abundance of fungal species known to be associated with respiratory diseases in indoor dust samples was similar, regardless of the visibility of fungi in the houses. The existence of fungi associated with respiratory symptoms was compared with several parameters like dust particulate matter (PM), CO_2_ level, temperature, and humidity. Most of these parameters are either positively or negatively correlated with the existence of fungi associated with respiratory diseases; however, none of these correlations were significant at *p* = 0.05. Our results indicate that implementing molecular methods for detecting indoor fungi may strengthen common exposure and risk assessment practices.

## 1. Introduction

The indoor environment is believed to have a significant health impact as people spend the majority of their time in a built environment. Past research has gone so far as to estimate that humans in industrialized countries spend up to 90% of their lives inside [1,2]. Several research articles have demonstrated the indoor microbial environment, specifically the microbial entities we contact primarily, like bacteria or fungi, and their health impacts on human occupants [3,4,5]. For example, an increased presence of fungi and bacteria in the home has been correlated with adverse health effects such as disease transmission, asthma, allergies, and flu-like symptoms [6,7,8]. In addition, various environmental factors (including temperature, moisture, and ventilation) can change the abundance and diversity of bacteria or fungi in the indoor microbiome. Likewise, an increase in humidity has been shown to impact fungal and bacterial abundance in indoor environments [9].

Emerging evidence suggests that damp houses favor the growth of fungal spores formed by *Penicillium*, *Aspergillus*, and *Cladosporium*, which may be associated with breathing problems [3,10]. The concentrations of fungal spores in the ambient air vary greatly depending on the environment, the climate, the time of day, and the season [11]. Fortunately, fungal contamination and dust mites can be controlled by reducing the humidity [12]. However, reducing humidity in homes with poor ventilation or humid climates has proven difficult. Fungi such as *Penicillium* sp., associated with asthma, and *Aspergillus* sp., associated with atopy, are the primary sources of fungal loads in these indoor spaces due to their ability to grow at high concentrations on substrates such as wallpaper or upholstery [11,13,14,15,16]. The global prevalence of “allergic fungal airway disease” (AFAD) is uncertain; however, it has been estimated that 4.8 million people worldwide with asthma have Allergic Bronchopulmonary Aspergillosis (ABPA), a pulmonary disease caused by *Aspergillus fumigatus* (AF) [17,18,19]. AF can cause a detrimental response in susceptible hosts, such as repeated wheezing and dyspnea, that can become life-threatening in acute cases [18,20]. In addition, AF-sensitized asthma can develop into ABPA via molecular allergens, as AF produces a multitude of factors, some of which can act as allergens that can aggravate asthma symptoms [18,21].

In the past, microbial analyses were predominately conducted by culturing. However, the vast majority (estimated at 99% or more) of microorganisms cannot be cultured [6]. It is also difficult to differentiate between species using culturing or direct visual observation-based methodologies due to morphological similarities. For fungi, research has traditionally focused on the visible detection of “molds” (which typically indicate only filamentous fungi) in homes, followed by microscopic identifications. However, the visual and microscopic identification method of fungi (or mold) is error-prone due to subjectivity and person-to-person variations [22,23]. It is also impossible to detect hundreds to thousands of different fungal taxa (often at species levels) by microscopic methods [24]. Moreover, there are no standard methods for fungal detection at the species level, nor are there any evaluation criteria for assessing fungal loads in indoor spaces [11]. These challenges call for more advanced approaches for a comprehensive look into the indoor microbiome of homes.

Molecular culture-independent approaches, such as quantitative polymerase chain reaction (qPCR), can assess the abundance and bypass the bias of morphological similarities in fungal communities [6]. However, PCR is limited to pre-determined target microorganisms and may not be suitable for the broad-spectrum detection of non-target microbes [25,26]. High-throughput sequencing methods, such as next-generation sequencing (NGS) and metagenomics approaches, overcome the limitations of traditional methods by offering target-independent identification of microbial taxa in complex samples [25,27]. Thus, by employing the NGS method, not only fungi but a diverse microbial community, including pathogens and hundreds or even thousands of other taxa, can be identified [28]. Furthermore, exposure to these microorganisms often occurs through airborne transmission. Therefore, assessment of the relative abundance of microorganisms is possible by analyzing dust particles to which the airborne microorganisms have attached [29].

In this study, we used NGS along with qRT-PCR to investigate the indoor microbiome, explicitly focusing on the fungal and bacterial communities of *homes with visible mold* (HVM) contamination and *homes with no visible mold* (HNM) in Memphis, TN, USA. Memphis is located near the running edge of the Mississippi River with humidity levels that often range between the 1950s and mid-1980s, which is 20% higher than the EPAs standards to reduce fungal presence [12], which makes Memphis a prime location to assess both fungal and bacterial communities. We address two specific questions in this study: (a) Is there any association between the diversity of fungi and bacteria communities found in homes? (b) Are there any differences in the relative abundance of pathogenic fungi found in homes with visible mold contamination vs. homes with no visible mold contamination?

## 2. Materials and Methods

### 2.1. Study Area and Sampling Plan

This study was performed in greater Memphis, TN, USA. Samples (described below) were collected using two recruitment methods. The first recruitment utilized the citizens’ mold remediation hotline of the Shelby County Health Department (SCHD). The second method utilized friends’ participation. The friend recruitment was based on known citizens in Memphis who requested participation because of fungal concerns and general air quality in their homes. These subjects did not have any visible fungi before or during the sampling period and therefore were categorized as the control group.

A total of twenty houses were sampled during the study. Ten of the twenty houses had visible fungi (H2, H3, H4, H5, H6, H7, H8, H9, H10, and H18), and ten houses did not (H1, H11, H12, H13, H14, H15, H16, H17, H19, and H20). Field sampling was a one-week test in the respective homes. Samples were taken from the home by vacuuming content, deploying environmental instruments/samplers, and filling out a field sheet and questionnaire. The instruments included a PM counter and a CO_2_ logger placed at a central location in the living room. During the home setup, the field sheet logged the date, time, location, and other important parameters. The questionnaire asked about relevant information used to gather specific data that could help the project’s objectives and aims. Samples were collected using an ORECK vacuum and respected filter attachment (DUSTREAM Collector) that captured the vacuumed dust content following the Vacuum Dust Sample Collection Protocol recommended by the U.S. Department of Housing and Urban Development (HUD) [30] with minor modifications. Briefly, dust content was captured from two different areas: air vents (return vents) and the ground. Air vents were vacuumed in the room or adjacent room of fungal growth (or the main living rooms and bedrooms for HNM) and categorized as short-term exposure. Ground samples were captured using the same method and categorized as long-term exposure. Based on the home setup, the number of air vents that were vacuumed ranged from 1 to 4. Ground samples were collected from either carpet or a hard floor. Carpet samples were vacuumed over a one-square-meter area. Hard floor samples were vacuumed in a two-square-meter area. Thus, each home provided two types of samples: one ground-collected sample and one air-vent-collected sample. The vacuum collection was conducted on the initial visit to the sampling home. The vacuum filters were transported to the lab and stored at 4 °C until extraction.

### 2.2. Measurement of Indoor Environment Parameters

The microbial community largely depends on different types of environmental factors, and thus we have measured dust particulate matter (PM), carbon dioxide (CO_2_), humidity, and temperature in all the sampled houses (both HNM and HVM). We have used a continuous PM counter (OPC-N2, Alphasense Inc., Great Notley, Braintree, UK) that could measure mass concentrations of small particles. Similarly, the CO_2_ datalogger (1% CO_2_ + RH/T Data Logger, Model: K-33 ELG, CO2Meter.com, Ormond Beach, FL, USA) could continuously measure the level of CO_2_ (ppm), humidity (%), and temperature (°C) using Gaslab software. The PM counter is already deployable after removing any excel sheets from its memory bank; no calibrations are needed for the PM counter as the software is built into the machine, whereas CO_2_ dataloggers were configured by clearing all previous logs, properly setting the time/date, and adjusting the collection interval to every 5 min.

### 2.3. DNA Extraction, Purification, and Sequencing

Genomic DNA (gDNA) was extracted from each sample (*n* = 40) using the Qiagen DNA PowerSoil kit, following the manufacturer’s protocol. Purification of the extracted DNA was performed using the Epigentek DNA Concentrator kit (P-1006). The V4 hypervariable region of the 16S rRNA of the metagenomic DNA was amplified using a set of universal primers 515F (5′-GTGYCAGCMGCCGCGGTAA-3′) and 806R (5′-GGACTACNVGGGTWTCTAAT-3′). Similarly, ITS1F (5′-CTTGGTCATTTAGAGGAAGTAA-3′) and ITS2R (5′-GCTGCGTTCTTCATCGATGC-3′) were used to amplify the ITS region of fungi. The forward primer was tagged with a barcode sequence. The PCR (28 cycles) was conducted with the HotStarTaq Plus Master Mix Kit (Qiagen, Germantown, MD, USA), following the manufacturer’s instructions. The confirmation of the amplification was conducted by checking the PCR product on a 2% agarose gel. In order to prepare the Illumina DNA library, an equal proportion of the PCR products (in terms of concentration and molecular weight) were pooled and purified by Ampure XP beads. The paired-end sequencing was conducted at MR DNA (Shallowater, TX, USA) on a MiSeq, following the manufacturer’s guidelines.

### 2.4. Sequence Processing and Analysis

The processing and analysis of the sequences were conducted following the pipeline described by Mukherjee et al. (2016) [31], with some modifications. In brief, the quality of both the sequence files was checked (Phred score 25 or >25), followed by the removal of barcodes and other short (<150 bp) sequences along with sequences with ambiguous base calls using fastp v0.23.0 developed and reported by Chen et al. (2018) [32]. The chimeras were removed from the obtained sequences and denoised using DADA2 [4]. The final fasta file containing the cleaned sequences was used to construct the Operational Taxonomic Units (OTUs) following the MR DNA analysis pipeline (MR DNA, Shallowater, TX, USA) [33] for the downstream analysis of the bacterial and fungal composition. The final fasta file containing the cleaned sequences was used to construct the Operational Taxonomic Units (OTUs) using the Ribosomal Database Project [34,35,36,37] for bacteria and the UNITE database for fungi and was clustered at a 3% divergence level (97% similarity). The generated BIOM file was used to calculate bacterial and fungal composition, and visualization was conducted in the Microbiomeanalyst platform [38] and R packages (ggplot and vegan).

### 2.5. PCR Detection of Fungal Species Known to Be Associated with Respiratory Complications

Fungal species targets were selected based on their inclusion in a panel called the Environmental Relative Moldiness Index (ERMI) –developed by the EPA- to evaluate mold issues in water-damaged homes. Most of the species selected in this study are from ERMI Group 1 and Group 2, known to be associated with allergic respiratory diseases, nosocomial infections, and allergic respiratory reactions [39,40,41]. They were detected using qPCR, considering the threshold cycle (CT) of 27 as the detection limit [42,43]. Each PCR mixture included the following: PCR master mix (12.5 µL), nuclease-free water (8 µL), primers (2 µL), and the respected DNA –home sample or positive control (2.5 uL) for a total value of 25 µL. The PCR conditions and primer sequences were according to previous reports by Haugland and Vesper [44] and Haugland et al. (2004) [45].

### 2.6. Statistical Analysis

The statistical analysis was conducted using R (Version 4.0.0, R Foundation, Indianapolis, IN, USA). R packages ‘Vegan’ [46] were used for ecological data analysis and visualization using ‘ggplots2’ [47]. We explored and visualized the differences in community patterns in houses with and without visible fungal growths using the two-dimensional (2D) nonmetric multidimensional scaling (NMDS) ordination method, in which a stress function assessed the goodness of fit of the ordination compared with the original sample ranking [48]. In this process, the OTU counts of fungi and bacteria (at the genus level) were rarefied. The OTU table was square-root transformed, and the ‘gower’ dissimilarity matrix was used as the best method for calculating the distance matrix for the gradient analysis. It was followed by quantitative evaluation by permutational multivariate analysis of variance (PERMANOVA) [49] with 999 permutations using the adonis function from ‘Vegan’ package to test if houses with visible fungal growth and houses with no-fungal growth harbor significantly different taxonomic compositions. The network plots are generated using a statistical software called PAST v 4.0 [50]. The Venn diagram is prepared using a web-based platform provided by Bioinformatics & Evolutionary Genomics (available at: http://bioinforma tics.psb.ugent.be/webtools/Venn/, accessed on 12 April 2023). All heatmaps are generated using TBtools software [51].

## 3. Results

### 3.1. Data Showed the Presence of Several Pathogenic Bacterial and Fungal Genera with Distinctive Patterns in the Ground versus Air Vent Dust

**Figure 1:**
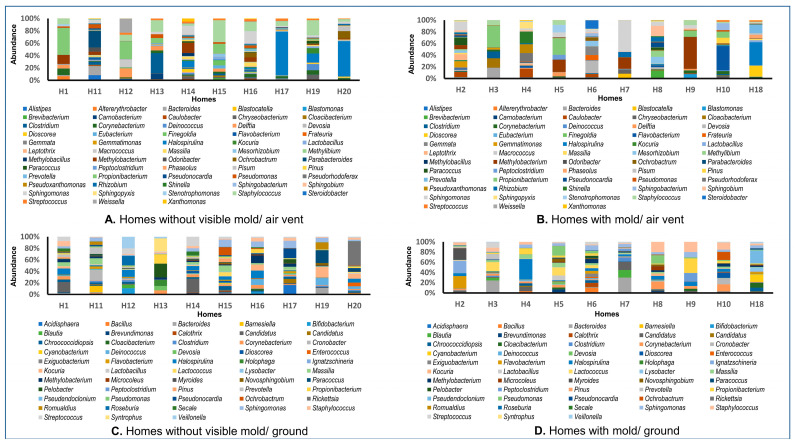
Diversity of bacterial genera in indoor dust samples collected from homes with or without visible mold (HVM and HNM) in Memphis. Panels (**A**,**B**) show the relative abundance (%) of air vent bacteria in HNM and HVM, respectively. The percent abundance of ground surface bacterial genera in HNM and HVM is presented in panels (**C**,**D**), respectively.

**Figure 2:**
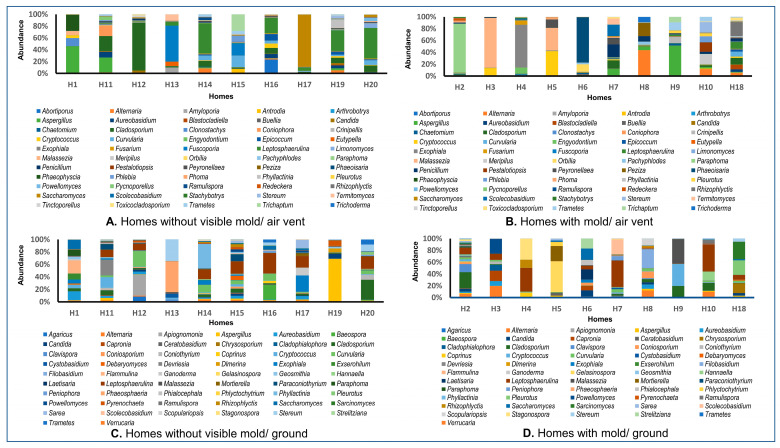
Diversity of fungal genera in indoor dust samples collected from homes with or without visible mold (HVM and HNM) in Memphis. Panels (**A**,**B**) show the relative abundance (%) of air vent fungal genera in HNM and HVM, respectively. Panels (**C**,**D**) represent the abundance percentage of ground surface fungal genera in HNM and HVM.

We have analyzed a total of 40 samples for both fungi and bacteria (ten samples each from the air vent and ground from HNM and ten samples each from the air vent and ground from HVM). The percentage abundance of bacterial and fungal genera in the air vent and ground samples of both HNM and HVM are shown in Figure 1 and Figure 2, respectively. The bacterial genera that were highly abundant across the air vent samples of HNM and HVM were *Propionibacterium*, *Sphingomonas*, *Staphylococcus*, *Corynebacterium*, *Methylobacterium*, and *Halospirulina* (Figure 1A,B). However, the most abundant bacterial genera in the ground surface samples of both HVM and HNM were *Streptococcus*, *Candidatus*, *Bacteroides*, *Sphingomonas*, *Massilia*, *Halospirulina*, *Staphylococcus*, and *Corynebacterium* (Figure 1C,D). Similarly, across HNM, the most abundant fungal genera in air vent samples were *Leptosphaerulina*, *Cladosporium*, *Curvularia*, and *Aspergillus* (Figure 2A,B). On the other hand, the fungal genera that were highly abundant on ground surfaces across HVM were *Leptosphaerulina*, *Cladosporium*, and *Aureobasidium* (Figure 2C,D).

The bacterial and fungal diversity in ground and air vent samples was measured using the Shannon diversity index (Figure S1). The comparison of bacterial diversity between ground and air vent samples in HNM and HVM is presented in Figure S1A,B, respectively. The result indicated that the ground samples have higher diversity in both house types compared to the air vent samples; however, the difference was not significant at *p* < 0.05. Similarly, the diversity of fungal communities in HNM and HVM was calculated and presented in Figure S1C,D, respectively. In both house types, the ground sample exhibited higher diversity compared to the air vent sample; however, none of these differences were significant at the *p* < 0.05 level.

**Figure 3:**
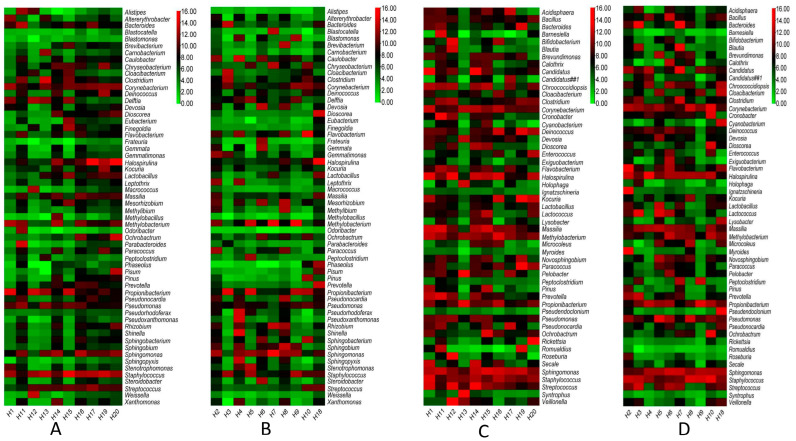
The heatmap demonstrates the abundance of the bacterial genera in the air vent and ground samples of both HNM and HVM. (**A**,**B**) represent the bacterial load in air vent samples of HNM and HVM, respectively. Panels (**C**,**D**) represent the bacterial load in ground samples HNM and HVM, respectively. All OTU values are log converted.

Based on the Operational Taxonomic Units (OTUs) data, a total of 58 and 53 bacterial genera were identified from the air vent and ground surfaces of both HNM and HVM and presented in Figure 3 using a heatmap diagram. The genus *Halospirulina* was observed to be the most abundant in the air vent samples of two HNM (H20 and H17, Figure 3A), while *Methylobacterium* was identified as the most abundant bacterial genera in the air vent sample of H8 (Figure 3B). The genus *Veillonella* was recorded to be the maximum in the ground sample of one HNM (H12, Figure 3C). However, in HVM (H2), the highest load of the bacterial genus belongs to *Ignatzschineria*, followed by *Flavobacterium* (Figure 3D).

**Figure 4:**
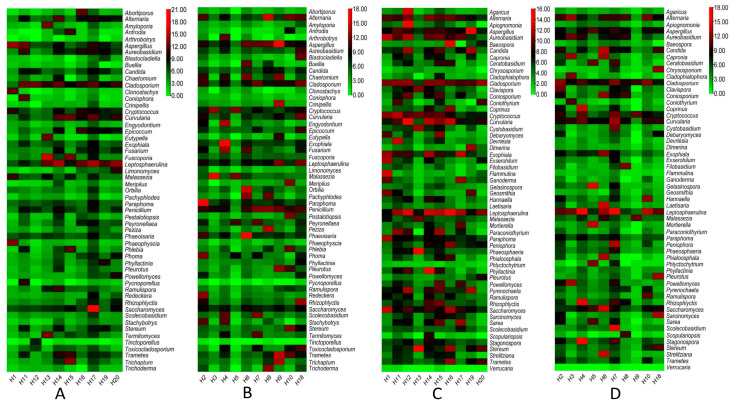
The heatmap demonstrates the abundance of fungal genera in the air vent and ground samples of both HNM and HVM. Panels (**A**,**B**) represent the fungal load in air vent samples of HNM and HVM, respectively. Panels (**C**,**D**) represent the fungal load in ground samples of HNM and HVM, respectively. All OTU values are log converted.

Similarly, a total of 91 fungal genera were recorded from OTU data and presented in Figure 4. In the air vent samples of both HNM and HVM, the maximum abundance of fungal genera belongs to *Saccharomyces* in H17 and *Phaeoisaria* in H6 (Figure 4A,B). At the same time, the maximum load of fungi in the ground samples of HNM and HVM belongs to the genera *Apiognomonia* and *Leptosphaerulina* (Figure 4C,D), respectively.

### 3.2. Network Analysis Shows Higher Variability in Fungal Than Bacterial Communities in Dust Samples from Air Vents and Ground

**Figure 5:**
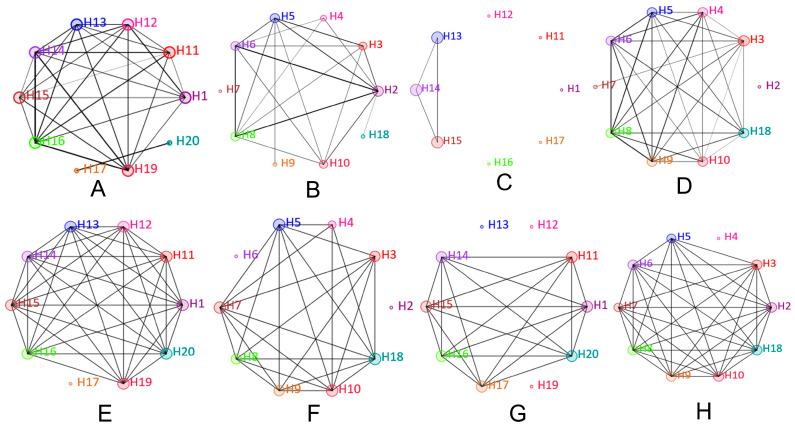
Network plots demonstrate the connection between houses based on bacterial and fungal genera’ composition. (**A**,**B**) represent the network connections of houses based on the bacterial composition of the air vent samples of HNM and HVM, respectively. Panels (**C**,**D**) represent the network connections of houses based on the bacterial composition of the ground samples of HNM and HVM, respectively. Panels (**E**,**F**) represent the network connections of houses based on fungal compositions in the air vent samples of HNM and HVM, respectively. Finally, panels (**G**,**H**) represent the network connections of houses based on fungal composition in the ground samples of HNM and HVM, respectively.

Network plots display the similarities in bacterial and fungal communities in HNM and HVM (Figure 5). The houses with similar bacterial and fungal genera’ compositions were connected with different numbers of nodes. However, there will be no network connection if the difference is significant. In the case of air vent samples collected from HNM and HVM, the composition of the bacterial candidates at the genus level shares similarities, except for H17 and H20 (Figure 5A) and H7 (Figure 5B), respectively. Interestingly, the bacterial communities in the ground samples obtained from HNM are less common compared to air vent samples (Figure 5C). However, in the case of ground samples obtained from HVM, the bacterial community composition is very close to each other except for H2 (Figure 5D). The fungal community compositions in air vent samples collected from HNM and HVM are nearly similar, except for H17 (Figure 5E), H2, and H6 (Figure 5F). In contrast, the fungal communities in the ground samples collected from H13, H12, and H19 (HNM) are not close to any other houses (Figure 5G). However, for ground samples collected from HVM, all the houses have similar fungal community compositions except for H4 (Figure 5H).

**Figure 6:**
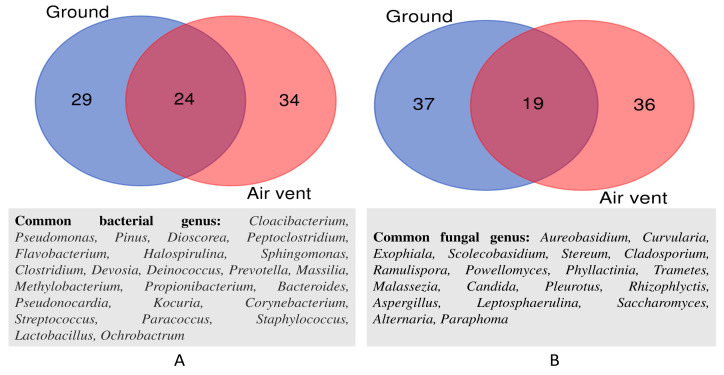
The Venn diagram demonstrates the unique and shared bacterial (**A**) and fungal (**B**) compositions between the ground and air vent samples collected from all twenty houses.

Furthermore, the bacterial and fungal genus composition differences in the ground and air vent samples were analyzed and presented in a Venn diagram (Figure 6). Ground and air vent samples contain 29 and 34 unique bacterial genera, respectively (Figure 6A); however, 24 bacterial genera such as *Cloacibacterium*, *Pseudomonas*, *Pinus*, *Dioscorea*, *Peptoclostridium*, *Flavobacterium*, *Halospirulina*, etc. are recorded to be common. Similarly, the unique fungal genus numbers are 37 and 36 in the ground and air vent samples, respectively. The shared fungal genera between these sample types belong to *Aureobasidium*, *Curvularia*, *Exophiala*, *Scolecobasidium*, *Stereum*, *Cladosporium*, *Ramulispora*, *Powellomyces*, *Phyllactinia*, *Trametes*, *Malassezia*, *Candida*, *Pleurotus*, *Rhizophlyctis*, *Aspergillus*, *Leptosphaerulina*, *Saccharomyces*, *Alternaria*, and *Paraphoma* (Figure 6B).

### 3.3. The Fungal Communities in Air Vent Samples Differ Significantly between Home Types

**Figure 7:**
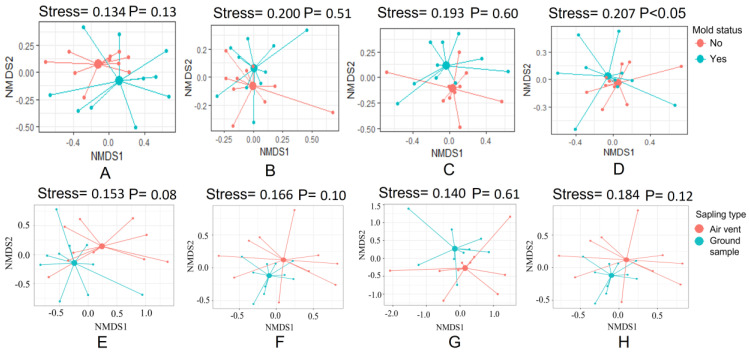
Nonmetric multidimensional scaling (NMDS) ordination plot presenting taxonomic composition differences observed in bacterial and fungal communities in HNM and HVM. Each data point in the plots represents the bacterial or fungal community (at the genus level) identified in a home. In (**A**) (air vent bacteria) and (**B**) (ground bacteria), red dots represent HNM, while blue dots represent HVM. In (**C**) (air vent fungi) and (**D**) (ground fungi), red dots represent HNM, while blue dots represent HVM associated with respiratory diseases in indoor dust samples collected from HNM and HVM. (**E**,**F**) represent the bacterial communities in ground and air vent samples in HNM and HVM, respectively. (**G**,**H**) demonstrate the fungal composition in ground and air vent samples in HNM and HVM, respectively.

To visualize the differences in taxonomic compositions between fungal and bacterial communities, we generated NMDS ordination plots (Figure 7), which provided information on observed fungal and bacterial communities (at the genus level) in HNM and HVM. No significant differences were observed between the air vent bacterial communities in HNM and HVM (Figure 7A, PERMANOVA, R2 = 0.07, *p* = 0.13). Similarly, no significant difference was observed among bacterial communities in ground samples collected from HNM and HVM (Figure 7B, PERMANOVA, R2 = 0.05, *p* = 0.51). No significant differences were observed in the ground dust samples from HNM and HVM (Figure 7C, PERMANOVA, R2 = 0.04, *p* = 0.60). However, a significant difference in fungal communities in air vent samples of HNM and HVM was observed (Figure 7D, PERMANOVA, R2 = 0.07, *p* < 0.05). Along with house types, the difference in the composition of bacterial and fungal communities in ground and air vent samples was also calculated. The difference in bacterial communities between ground and air vent samples in HNM (Figure 7E) and HVM (Figure 7F) showed a good fit (stress 0.153 and 0.166, respectively) but was not significant at the *p* < 0.05 level (R2 = 0.07 and 0.06, respectively). Similarly, the difference in fungal communities between ground and air vent samples in HNM and HVM was presented in Figure 7G,H, respectively. In both houses, the difference in fungal compositions in ground and air vent samples was not significant at the *p* < 0.05 level (R2 = 0.04 and 0.06, respectively).

### 3.4. Respiratory Disease-Related Fungal Species Were Detected in Both Home Types, Regardless of the Visibility of Fungi (Molds) in the Home

**Figure 8:**
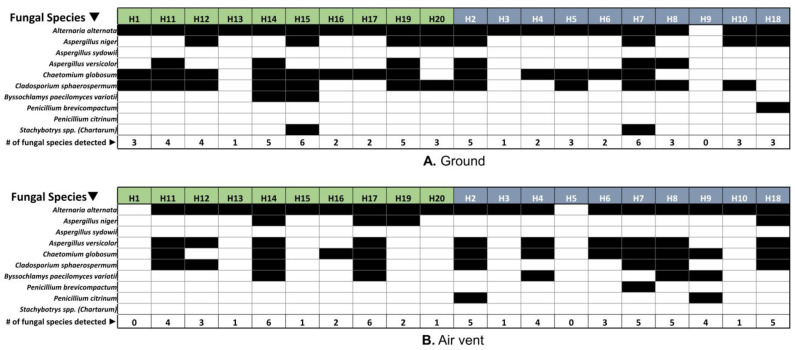
The checkered box demonstrates the presence of selected fungal species known to be associated with respiratory diseases in both HNM and HVM enumerated by qPCR. Black (present), white (absent). (**A**,**B**) indicate the ground and air vent samples.

The presence and absence of a selected group of fungi known to be associated with respiratory complications [39,40,41] have been screened using qPCR from indoor dust samples and presented in Figure 8. The abundance of fungal species known to be associated with respiratory diseases is comparable, regardless of the visibility status of fungi/mold in the home. *Alternaria alternata* was recorded as the most abundant fungal species in ground and air vent dust samples from both HNM and HVM, with some exceptions. For example, it was not detected in the ground sample collected from H9 or the air vent samples collected from H1 and d H5. *Aspergillus niger* and *Aspergillus versicolor* were also found in some houses’ ground and air vent samples; however, *Aspergillus sydowii* was not recorded in any of those houses.

### 3.5. Respiratory Disease-Associated Fungi Did Not Show Any Significant Correlation with Indoor Environmental Parameters

Furthermore, to find the relationship between fungal communities known to be associated with respiratory disease and the indoor environment, we measured indoor environmental parameters, including particulate dust matter, CO_2_ concentration, temperature, and relative humidity. The relationships were examined using Spearman correlations for ground (Table 1) and air vent samples (Table 2). The result depicted a weak positive correlation between all indoor parameters and fungal communities in the ground samples collected from HNM (Table 1). However, the correlation was negative in ground samples collected from HVM, except for PM10 (Table 1). Similarly, in the air vent sample, the correlation was positive for PM, CO_2_ level, and humidity and negative for temperature (Table 2). In HVM, the correlation was negative between fungal communities and indoor parameters like PM and CO_2_ levels. However, temperature and humidity exhibited a very weak positive correlation (Table 2). Overall, all the correlations were not statistically significant at *p* < 0.05.

## 4. Discussion

Microorganisms are ubiquitous in environments, including indoor buildings. Phenotype-based identification has been successfully used to identify fungi in the past but can be unhelpful for atypical morphologies or fungi that fail to sporulate. It is time-consuming and laborious. Rapid identification of molds can facilitate a more timely and accurate assessment of exposure to pathogenic fungi. Thus, our study used molecular approaches to identify and enumerate the bacterial and fungal communities found in HNM and HVM. In the past, few studies have explored microbial composition in homes and home appliances like freezers, TVs, pillowcases, kitchen cutting boards, etc. [3,28,52]. Interestingly, the microbial composition and diversity (mostly bacterial and fungi) vary significantly from one house to another depending on the place, latitude, and humidity [52]. Several previous studies have reported variability in indoor fungal diversity based on factors such as house types, sampling types, indoor household locations, and items sampled [53,54,55]. In this study, we collected dust samples from different houses in Memphis, TN, USA, and explored the bacterial and fungal diversity and abundance. We found that the diversity of fungal communities varied in contrast to the consistency of bacterial communities. Given the geographic location of Memphis-Shelby County and the history of the high prevalence of pediatric asthma and other allergic diseases in this metropolitan area [56,57], our study provides the first analytical information on the indoor fungal and bacterial loads in this region of the USA.

In our experiment, the bacterial genere *Rickettsia* and *Halospirulina* were found to be common in both HNM and HVM. The genus *Rickettsia* is a Gram-negative, non-spore-forming bacteria and is reported to be responsible for causing spotted fever and typhus in humans [58]. Conversely, information regarding the interaction of *Halospirulina* with humans is lacking. In HNM, one of the most abundant genera was *Streptococcus*, while in HVM, *Staphylococcus* was abundant. This agrees with several earlier studies showing that *Streptococcus* and *Staphylococcus* are the two most abundant bacteria found in indoor environments [59,60,61].

The most abundant fungal genus in all these houses was found to be *Leptosphaerulina*. The genus *Leptosphaerulina* comprises roughly twenty-five species of filamentous ascomycetes that produce dark-colored pseudothecia and are primarily associated with plant diseases [62,63]. It may be noted that the ascospores of *Leptosphaerulina* are rarely reported from indoor air samples. On the other hand, its anamorph, *Pithomyces* [64], is common in the outdoor air worldwide, including in two North American cities (New York and Toronto) [65]. Similar to our results, *Leptosphaerulina* was found to be one of the most abundant genera in a previous study utilizing culture-independent molecular methods to evaluate the effect of water damage in buildings and subsequent renovation/remediation on indoor fungal communities [66]. On the other hand, *Alternaria alternata*, a major allergen and one of the most common fungi often associated with asthma development, persistence, and exacerbation [67,68,69], was found abundant in HNM and HVM. In addition, *Chaetomium globosum* (abundant in both home types) is a fungus with type I and III allergens associated with skin and nail infections in humans and infrequently causes cerebral and systemic infections in immunocompromised individuals [70,71,72,73]. In a pilot study, Andersen et al. investigated the air quality in different houses (with no visible fungi) in Denmark. They reported that *Alternaria*, *Pseudopithomyces*, and *Cladosporium* were the dominant fungus genera in an indoor environment, which is consistent with our finding [74]. *Alternaria*, *Cladosporium*, and *Leptosphaerulina* (*Pithomyces*) are common outdoor fungi; thus, they may have originated and been transmitted from outdoor to indoor environments. Interestingly, in our metataxonomic analyses, the abundance of *Penicillium* was found to be low, which is contrary to several previous reports showing *Penicillium* as one of the most abundant airborne fungal genera in the indoor air of moldy homes [9,53,74,75]. However, our qPCR assays detected *Penicillium brevicompactum* in the ground and air vent samples from at least one moldy home, while air vent samples from two moldy homes yielded positive results for *Penicillium citrinum*. The low detection rates of *Penicillium* could be attributed to the assay design (NGS versus qPCR), differences in environmental conditions, or other unknown factors.

The NMDS gradient analysis produces ordinations based on the dissimilarity matrix, or distance between different data sets. In this investigation, the NMDS analysis (Figure 7) revealed that the composition of bacterial communities in HNM and HVM is similar. This agrees with past studies that found bacterial communities differ depending on the surface type [2,8,28,76]. Although the NMDS analysis displayed no significant differences in ground fungal communities regardless of visible fungal presence or absence in homes, there is a significant difference in fungal communities in air vent samples collected from HNM and HVM. Visible fungi indicate an active fungal (“mold”) problem; however, there is also a chance of a nonvisible prevalence of pathogenic fungi in homes. This further confirms the importance of analyzing fungal diversity in homes in order to detect hidden mold using adequate techniques, e.g., molecular approaches [24,25,77].

The diversity of microbial communities in an indoor environment highly depends on the building’s occupants and the surrounding outdoor environment [78]. There is always an exchange between indoor and outdoor environments through human activities, water through the plumbing system, and air passages (windows, ventilation system, heating system, etc.), which determine the shape of the microbial community. Furthermore, humidity varies significantly from one season (winter) to another (summer) and is considered a critical regulatory factor in airborne microbial (especially fungi) growth and pathogenic agents [78]. In this investigation, we have tried to correlate the fungal species that are known to be associated with respiratory illnesses with their indoor environments, like dust particle size (PM), CO_2_ level, humidity, and temperature, using Sperman’s Rho statistics. Depending on the house status (HNM and HVM) and sampling site (ground and air vent), fungal communities exhibited some positive and negative relationships; however, none of these correlations were statistically significant (*p* < 0.05). This could be due to a number of factors, such as sample size, variability in environmental conditions, or other unknown factors that may have influenced the results. Environmental factors are known to be associated with asthma and other respiratory illnesses [79]. To this end, the agents, fungi, or bacteria that may prompt adverse health reactions need further consideration. Indoor fungal exposure has been studied countless times with scientific data backing it, acknowledging that exposure can lead to increased severity of asthma, as shown by human intervention and mouse trials [7,80].

Exposures to pathogenic fungi, including molds, are reported to be associated with several health problems, such as allergies, shortness of breath, chest tightness, and other respiratory complications [40,41]. Using qPCR analysis, a previous study investigated the occurrence of ten variable fungal and three bacterial groups in about 3000 homes in different geographical locations [52]. Their result demonstrated that the abundance of fungal and bacterial candidates largely depends on geographical location. In this study, we have assessed the presence of fungal targets from Group 1 and Group 2 of the ERMI-panel (developed by the US EPA) and fungi that are reported to be associated with allergic respiratory diseases, infections, and allergic respiratory reactions in both house types (HVM and HNM) using qPCR (Figure 8). The most common fungi found in both ground and air vent dust samples in both house types were *Alternaria alternata*, followed by *Chaetomium globosum*. The spore produced by *A. alternata* is a well-known biological contaminant and has been reported to be an inducer of several respiratory illnesses [81]. *Chaetomium globosum* is a mesophilic mold ubiquitously distributed in nature and was reported to be associated with onychomycosis [82]. This filamentous indoor fungus is often found on damp gypsum boards or plywood [83,84]. Therefore, *Chaetomium* abundance in both house types (moldy and non-moldy) in the present study may indicate the existence of dampness on gypsum boards or plywood wallboards. Furthermore, *Aspergillus versicolor* was also detected in many homes (both HVM and HNM), which is associated with several health problems, including neurological issues and pulmonary infections [85,86]. Interestingly, we have detected a broad spectrum of fungi associated with several respiratory complications in homes designated as “no visible mold” (HNM). Furthermore, there was no distinct demarcation in response to the presence and absence of mold species associated with health issues between HVM and HNM. So, our observation strongly suggests that homes without visible mold are not risk-free and may induce health complications. This study provides two new contributions to our understanding of the attribution of microbial risk factors in indoor environments. First, by employing genomics/sequencing-based methods, we empirically proved that molecular techniques that utilize the detection of microbial nucleic acids and genetic materials provide a significantly higher resolution in identifying potential microbial hazards in subjects that the visual and qualitative identification modalities failed to uncover. We unearthed how non-moldy homes may pose “hidden” risks of pathogenic microbial exposures, which could be statistically similar to the ones from moldy homes. Second, our study contributes to the information on indoor microbial (fungal and bacterial) prevalence information, for the first time, from a metropolitan area in a geographic location (mid-south US) that reports higher rates of pediatric asthma and other respiratory diseases, coupled with poor housing conditions [56,57].

The limitations of the present study may include a lack of statistical power as we only sampled from a total of twenty homes. Nonetheless, there are reports of similar studies with a limited sample size (such as *n* = 28 homes) [75]. A larger sample size (home samples) and repeated sample collections might be valuable to establish a significant correlation between fungi status and indoor environments. However, despite the limited number of homes sampled, we could display in-depth molecular profiles of bacteria and fungi in these homes, showcasing the importance and utility of molecular methods. Moreover, our results indicate that fungal exposure does not depend on the presence of visible fungi/mold in a home. This means the exposure may also come from “invisible” fungi/molds, such as microscopic fungi spores, as reported previously [87].

## 5. Conclusions

In this study, we used molecular methods to report the bacterial and fungal communities, the presence of fungal species known to be associated with respiratory diseases in HNM and HVM, and their correlations with indoor environmental factors. We found that these molecular methods provide rich information and are very useful in fungal and bacterial diagnostics in indoor environments. Future research aimed at assessing indoor air quality in homes will need to use updated technologies to ascertain the correlation between the microbiota of the built environment and adverse health effects.

## Figures and Tables

**Table 1:** Spearman correlations of OTUs of fungal species known to be associated with respiratory diseases in ground dust samples with multiple indoor environmental parameters.

House Type	Correlation Between	Correlation Coefficient, r_s_	Correlation	*p* Value
Houses with no visible mold (HNM)	OTU: PM 1.0	0.22	Positive	0.53
	OTU: PM 2.5	0.22	Positive	0.53
	OTU: PM 10.0	0.23	Positive	0.51
	OTU: CO_2_ level	0.35	Positive	0.38
	OTU: Humidity	0.28	Positive	0.49
	OUT: Temperature	0.38	Positive	0.27
Houses with visible mold (HVM)	OTU: PM 1.0	−0.05	Negative	0.87
	OTU: PM 2.5	−0.05	Negative	0.87
	OTU: PM 10.0	0.01	Positive	0.96
	OTU: CO_2_ level	−0.28	Negative	0.42
	OTU: Humidity	−0.03	Negative	0.93
	OTU: Temperature	−0.21	Negative	0.55

**Table 2:** Spearman correlations of OTUs of fungal species known to be associated with respiratory diseases in air vent samples with multiple indoor environmental parameters.

House Type	Correlation Between	Correlation Coefficient, r_s_	Correlation	*p* Value
Houses with no visible mold (HNM)	OTU: PM 1.0	0.62	Positive	0.05
	OTU: PM 2.5	0.62	Positive	0.05
	OTU: PM 10.0	0.46	Positive	0.17
	OTU: CO_2_ level	0.23	Positive	0.57
	OTU: Humidity	0.30	Positive	0.45
	OTU: Temperature	−0.22	Negative	0.52
Houses with visible mold (HVM)	OTU: PM 1.0	−0.48	Negative	0.17
	OTU: PM 2.5	−0.48	Negative	0.17
	OTU: PM 10.0	−0.45	Negative	0.19
	OTU: CO_2_ level	−0.45	Negative	0.18
	OTU: Humidity	0.10	Positive	0.77
	OTU: Temperature	0.11	Positive	0.75

## Data Availability

This manuscript contains metagenomic sequence data from both bacteria (40) and fungi (40). The metataxonomic sequence datasets associated with this paper are archived at the NCBI Sequence Read Archive (SRA) under the BioProject accession number PRJNA816110.

## Supplementary Materials

The following are available online at https://www.mdpi.com/article/10.3390/pathogens12081006/s1, Figure S1: Represents the Shannon alpha diversity in HNM and HVM. (A) and (B) indicate the bacterial diversity in ground and air vent samples in HNM and HVM, respectively. (C) and (D) demonstrate the fungal diversity in ground and air vent samples in HNM and HVM, respectively.
