# Supplementary material for: Indoor Bacterial and Fungal Burden in “Moldy” versus “Non-Moldy” Homes: A Case Study Employing Advanced Sequencing Techniques in a US Metropolitan Area
Source: Pathogens. 2023 Aug 1;12(8):1006. doi: 10.3390/pathogens12081006 (PMC10457890; doi:10.3390/pathogens12081006)
Supplement: Supplementary file 1 [file pathogens-12-01006-s001.zip › pathogens-2465356-supplementary.pdf]

The alpha diversity

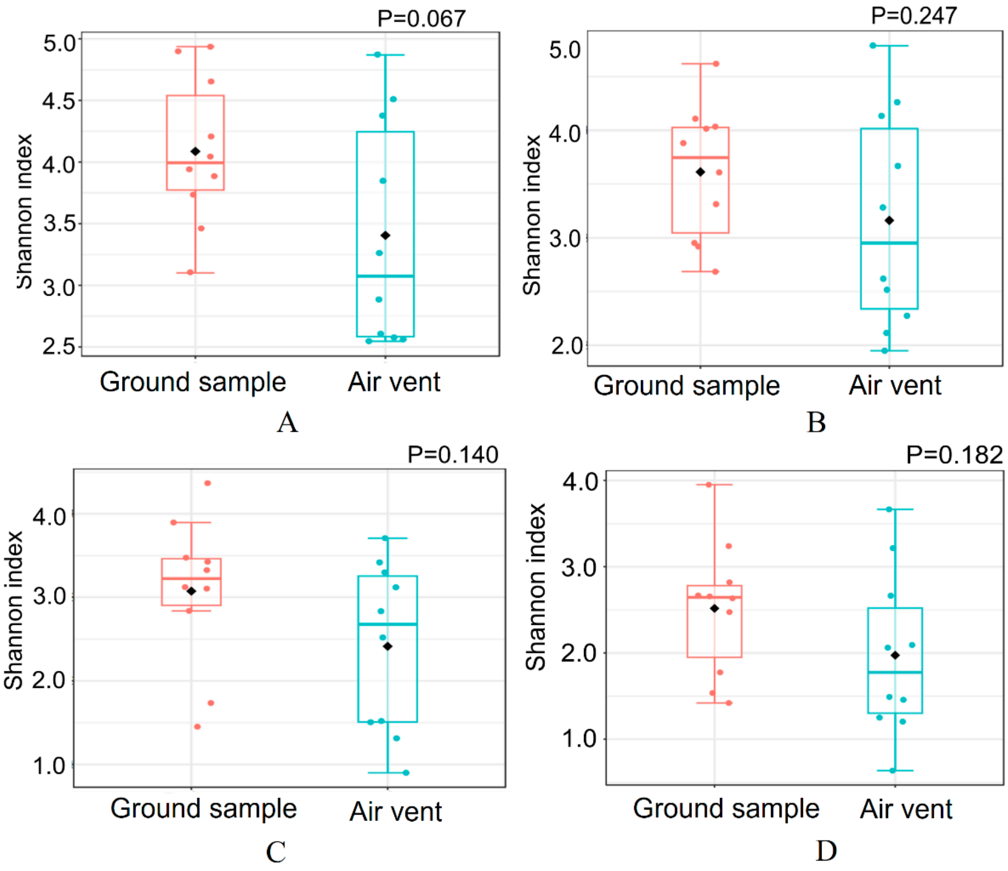

Figure S1. Represents the Shannon alpha diversity in HNM and HVM. (A) and (B) indicate the bacterial diversity in the ground and air vent samples in HNM and HVM, respectively. (C) and (D) demonstrate the fungal diversity in the ground and air vent samples in HNM and HVM, respectively.
